# An intelligent cluster optimization algorithm based on Whale Optimization Algorithm for VANETs (WOACNET)

**DOI:** 10.1371/journal.pone.0250271

**Published:** 2021-04-21

**Authors:** Ghassan Husnain, Shahzad Anwar

**Affiliations:** 1 Department of Mechatronics Engineering, University of Engineering and Technology, Peshawar, Pakistan; 2 Department of Computer Science, Iqra National University, Peshawar, Pakistan; Torrens University Australia, AUSTRALIA

## Abstract

Vehicular Ad hoc Networks (VANETs) an important category in networking focuses on many applications, such as safety and intelligent traffic management systems. The high node mobility and sparse vehicle distribution (on the road) compromise VANETs network scalability and rapid topology, hence creating major challenges, such as network physical layout formation, unstable links to enable robust, reliable, and scalable vehicle communication, especially in a dense traffic network. This study discusses a novel optimization approach considering transmission range, node density, speed, direction, and grid size during clustering. Whale Optimization Algorithm for Clustering in Vehicular Ad hoc Networks (WOACNET) was introduced to select an optimum cluster head (CH) and was calculated and evaluated based on intelligence and capability. Initially, simulations were performed, Subsequently, rigorous experimentations were conducted on WOACNET. The model was compared and evaluated with state-of-the-art well-established other methods, such as Gray Wolf Optimization (GWO) and Ant Lion Optimization (ALO) employing various performance metrics. The results demonstrate that the developed method performance is well ahead compared to other methods in VANET in terms of cluster head, varying transmission ranges, grid size, and nodes. The developed method results in achieving an overall 46% enhancement in cluster optimization and an F-value of 31.64 compared to other established methods (11.95 and 22.50) consequently, increase in cluster lifetime.

## 1. Introduction

In the last few decades, meta-heuristic approaches are getting popular in the area of computer vision and machine learning. These famous meta-heuristic approaches include Particle Swarm Optimization, Genetic Algorithms, and Ant Colony Optimization (ACO). These meta-heuristic approaches act a vital part in computer science and related areas. This raises some questions that why these algorithms are becoming more common as compared to other algorithms in these fields. Researchers suggest that flexibility, simplicity, local optima avoidance, easy understanding, and deviation-free approaches are the reason behind their common usage. These algorithms are flexible enough to apply to the problems of different natures which is the main reason behind their increased usage. These algorithms have a lenient nature which makes their applicability easy to implement. Many meta-heuristic approaches are deviation-free and use variables randomness to solve different problems. These methods start with a random solution, excluding the calculations for the deriving of finding space which makes it suitable to solve present problems. These solutions are generally inspired by nature, animals, insects, and birds. Therefore, these algorithms are easy to understand and easily extendable. Lastly, these algorithms focus on exploring the whole working space eliminating the local optima problem which is unsuitable for any kind of problem. IoT being an important field of networks acts as a significant part of everyone’s lives and it is increasing and modernizing every day for a prosperous future [1]. The IoT is a composition of different types of networks. Considering the aforementioned issues, intelligent clustering algorithms can play an important role for VANETs by making them more manageable, scalable, optimized, and by balancing network load. Clustering means grouping or collection of nodes and one of the nodes is designated as CH or cluster node. Network clustering means the grouping of nodes using their similarities. The similarity between nodes can be measured by the distance between nodes and the availability of bandwidth, speed, and direction of vehicular nodes. Different clustering algorithms differ from each other based on some grouping rules. The cluster size in VANET depends on the transmission range of the vehicular node. The cluster which comprises of vehicles will be directly relational to the communication limit of those nodes. While creating these clusters considering other important parameters like transmission ranges, grid size, and the number of nodes, speed, and direction of nodes are also very important as the lifetime of these clusters be increased directly and the overall performance of the entire network could be optimized indirectly. In this scenario, an intelligent node clustering is required which offers a minimum number of clusters, CHs, and long life of clusters. This will reduce the communication cost for the system by minimizing the sum of the cluster to near optimum and increased cluster lifetime. This will reduce the requirements of the resources in VANETs which will eventually increase the network lifetime. The more time the nodes spend in a cluster the better will be the networks’ enactment. The network nodes’ clustering is an NP-hard problem and s/election of CHs acts a vital role in this clustering process. The role of CH includes the formation and end of the clusters, topology selection for maintenance, and resource provision to cluster members. CH also manages the communication for both within the cluster and with other available clusters in the network. The network performance in this scenario can be considered by the clustering stability which can be measured by the CH change ratio and conversion ratio of cluster nodes to CH. Therefore, it is necessary to optimize the CHs for intermittent connectivity and efficient data dissemination. The optimized clusters provide high throughput, reliability, and low endways communication latency among vehicles. In addition, the VANETs is more scalable for optimized CH’s. In this research, the objective is to optimize a number of clusters to dynamic transmission range, network nodes and grid sizes. Furthermore, WOACNET is mathematically modeled to achieve the optimization of vehicular clusters. To the best of information, this is a vital attempt to implement the WOACNET algorithm for vehicular clustering. Literature has various clustering algorithms for optimizing the performance of VANETs, including clustering but there is still space to optimize the clustering process for enhancing the overall network performance. Moreover, Optimization Challenges carry excellent implications within the scientific engineering model along with decision-making application. The term optimization is used for many counteragents of an issue. Where it will conform to the extreme value associated with more than one objective. If an optimization problem carries just one objective the option of choosing the best outcome is known as single-objective problem. Generally, in a single-objective problem, the focal point is on procuring just a single solution with exception of multimodal function. If the optimization problem has considerable objective functions the optimization is referred to as Multi-Objective Problem (MOP). Here and now the majority of issues belong to MOPs as they environ a variety of objectives which then require to be optimized simultaneously. Clustering in VANET is also another predicament in MOPs [2]. Many traditional mathematical programming paths produce a single solution in MOPs Hence consequently such approaches may not be pertinent to enhance MOPs. The metamorphic authorisms paradigm is rather desirable to fix MOPS as they are population-based, which as a result facilitates them to construct a group of solutions in a single iteration. In multi-objective optimization problems (MOPs) there are transformative algorithms designed and are credible for retrieving numerous solutions. These algorithms are designed for achieving multiple solutions in a given time instead of just one solution. Many evolutionary algorithms have been developed, they perform different mechanisms to acquire the solution, for example, genetic algorithm, differential evolution, artificial immune system, and swarm intelligence [3]. So that was the reason to propose a novel evolutionary algorithm Multi-Objective Whale Optimization Algorithm for clustering in VANETs. In this study, Multi-objective WOA (MOWOA) has been revisited for cluster optimization. Initially, a search phase for vehicles has been proposed via employing self-adaptive weights minimizing the error. Subsequently, Cluster Head selection criteria based on fitness function were performed for network management. These two functions are used to improve the accuracy of MOWOA. The main contributions are:

- Mathematically modeling of a novel WOA algorithm for optimization of clusters in VANETs.
- Multi-objective clustering with help of weight assignment to each objective as per network/user requirement.
- A thorough comparative analysis, by applying different evaluation measures like Load Balance Factor (LBF), grid size, and a number of cluster heads to confirm the advantage of the developed solution as compared to the traditional schemes.

In this study, WOA method has been revisited/ modified resulting in proposing and developing a novel WOACNET as per the requirements of clustering optimization in Vehicular Ad Hoc Network for Intelligent Transportation to reduce the number of clusters hence increasing the network lifetime.

The remaining of the article is organized as follows: In section 2, issues and challenges in the development of VANET routing protocols, and different categorize of VANET routing protocols are presented. The proposed method is described in section 3 and section 4 shows the experimentation results followed by discussion. Finally, section 5 concludes this study.

## 2. Literature review

VANETs have many applications in the literature such that real-time safety applications, mobile-health systems, smartphone-based methods, autonomous vehicles, wearable sensor-based methods, systems for clustering vehicles using VANETs [1]. Numerous protocols have been designed for Wireless Sensor Networks (WSN) and Mobile Ad hoc Networks (MANETs) [4]. However, in the case of VANET, a set of rules designed for WSN and MANETs are less implemented due to the special nature of VANETs.

The improvement in routing protocols for VANETs is a challenging task due to its unique specifications, some of the key design challenges are a dynamic network of topology, heterogeneity of devices, transmission range, node density, and privacy and security. For efficient communication systems, the proper network topology is required as it affects the communication between nodes. It is important specifically for VANETs because of the short transmission range and frequent motion of vehicular nodes. In literature, two approaches are deployed i.e.; single-hop and multi-hop communication. In the former approach, packets are transmitted straight from every device to the destination. While in later approach clustering technique is incorporated optimizing multi-hop communication in VANETs [5]. In VANETs nodes are heterogeneous so are their specifications such as memory consumption and power usage, which leads to challenges such as the Quality of Service (QoS), etc. [6].

Bio-enlivened organization specialized techniques utilize computational strategies to tackle correspondence issues. The fundamental reason utilized by these techniques is to emulate the common conduct of living things, (for example, people, bugs, creatures) as they attempt to discover answers for their normal necessities, (for example, food, propagation, self-preservation, versatility, and so forth). From the past many years, the rise of bio-motivated schemes introduced considering various correspondence (in zones), for instance, course, gridlock, security, and so forth. The fundamental inspiration for the transmission of bio-roused correspondence strategies is from the solid likenesses between correspondence conditions in correspondence and the normal association of species. Next, an evaluation of the existing topologies makes a bio-inspired protocol a feasible approach.

As far as the enormous amount of devices in Wireless Sensor Networks (WSNs) information assortment, stays a significant test [7, 8]. To address the issue of information procurement on WSNs, [9] have proposed the utilization of cell sinks (certain hubs are liable for getting and handling network information to help settle on choices applicable to the organization setting: woodland, ranch.) In [9], proposed a re-enactment law called SIMPLE roused by Swarm Intelligence, to draw out organization life. Since SIMPLE depends on swarm intelligence, therefore worldwide organization data to improve network wellbeing is not required. Durable conduct between hubs (otherwise called coordinated conduct) is intended to accomplish the full usefulness of the earth. Basic has been appeared to show better distortion and heartiness contrasted with hub disappointment contrasted with Ad hoc On-request Distance Vector steering (AODV) [10], Dynamic Source Routing (DSR) [11], and customary course calculation called the Max-min Remaining Energy Protocol (MREP) [12].

In [18], the writers utilized the naturally inspired calculation Particle Swarm Optimization (PSO) to improve the MANET multicast course. PSO has been utilized effectively in [20] to tackle the Multicast Routing Problem (MPR) where the most developed courses are required. The outcomes acquired in [21] show an intricate decrease regarding more limited multicast access pathways contrasted with GA calculation. Based on the literature review several routing algorithms for VANET routing have been proposed. These algorithms may be classified as transmitting practices based on cross layers, transmitting rules based on the quality of service, and channeling rules based on clustering. Cluster-based routing protocols consist of network protocols that incorporate cluster formation. All the vehicular devices in the system are mapped into different clusters. One node in each cluster is given the responsibility of collecting data from all nodes in that specific cluster and transmit to the other clusters or sinks. This primary node with responsibilities is called cluster head (CH). CHs are selected through some metrics. CH is used so that direct communication links from sensing nodes to the sink of data can be reduced. However, the selection of CH is a major overhead [15].

The use of the evolutionary algorithms for clustering in other types of ad hoc networks is also very popular. For instance, in wireless body area network [22], proposed a technique named Ant Colony Optimization that tends to decrease the number of shortest communication links to the sink. Previous approaches to this algorithm supposed that all sensing nodes must be in the vicinity of the sink for communication, but this problem is solved by this algorithm by introducing CHs in each cluster. Reliability and energy consumption are not considered in this approach. Melody et.al in [13], proposed another improvement in this domain named Hybrid Indirect Transmission (HIT), which shows that HIT provides high energy efficiency, low network delay, and a high lifetime of the network. Both cluster-based routing algorithms for WBAN provide a reduction of power consumption by reducing the number of communication links. The main advantage of Ant Colony Optimization [22] is that number of clusters remains constant even number of nodes is increased. A study presented in [14] a swarm-based routing optimization technique for VANETs, they incorporate an ACO-based approach for routing optimization. A comparative analysis of modern and advanced techniques is presented in [15], for various experimental scenarios. Some critical parameters are neglected in this work. Some recent studies [16–20] proposed a similar optimization, based on evolutionary algorithms namely Dragonfly, GWO, CLPSO, MOPSO, and ALO, MOPSO in [21], ACO in [22], GWOCNET in [23], and CAVDO in [24]. These methods are based on evolutionary computations, moreover, the highway scenario is considered in all this literature. Routing optimization based on evolutionary or genetic algorithms is also proposed for other types of ad hoc networks such as similar techniques for Flying Ad hoc Networks and are available in [25, 26] and in [27] for body area networks. Some recent work on clustering in VANETs has been proposed in [28], where a K-Harmonic means clustering technique is introduced to improve communication links between vehicles. A recent study discusses population size which is usually set between 20 and 100 in bio-inspired clustering, considering only nodes for communication that have relatively high energy as compared to other nodes in a networking group [29]. In [30], an enhanced Particle Swarm Optimization (PSO) method for clustering is introduced by considering only those nodes which are in the same direction and same velocity. Quality of Service (QoS) and identification of malicious nodes for improved clustering in VANETs have been proposed in [31]. A new approach [32] using Software Defined Network (SDN) has been proposed to optimize cluster heads in an urban area. A new fuzzy logic-based clustering scheme has been proposed in [33]. Authors in [34], have proposed a game theory-based clustering method in VANETs that aims to provide uninterrupted connection amongst the nodes. A secured clustering technique based on a cryptography scheme for the urban area has been proposed in [35]. A new application in VANETs for video streaming using enhanced Quality of Service (QoS) has been proposed by authors in [36].

## 3. Material and methodology

Generally routing protocol may not have the capability to cover and address all the vital parameters for communication, rather focus on few specific parameters. For instance, routing protocols based on temperature rise mainly focus on the reduction of temperature of nodes and select routes based on hotspots and avoiding motion of the body and energy efficiency. The case with all other routing protocols that they omit some key parameters of VANET and focus on a specific one. The demand for the development of improved routing protocols is vital, considering most of the key parameters of VANET communication. The problem addressed in this paper is to develop an innovative, intelligent WOACNET considering many parameters simultaneously, finding an efficient solution that incorporates all the challenges and issues of VANET. In the developed framework, an intelligent clustering approach is employed to optimize the routing of data packets throughout the VANET so that network becomes more optimized, manageable, and scalable. Clustering in VANETS based on some similarities and dissimilarities, Vehicular nodes are grouped to accomplish some specific goals. Some parameters are used to judge the similarity and dissimilarity of nodes like direction, speed, the distance among nodes, and transmission range. The CH has responsibilities like cluster formation, gathering data from all nodes within the cluster, the transmission of that data to other CHs, efficient routing of data packets within and outside the cluster, supplying resources to the member nodes, network maintenance, and termination of the cluster.

In contrast to the clustering approach, if other routing approaches are analyzed in which every node directly communicates to the external server or side unit used in the infrastructure, communication may be impaired. More specifically if the crowded environment (highways, congested roads) is concerned in which a lot of vehicular nodes try to access the network resources, and the network accumulators may be chocked due to heavy traffic. This is because every node is sending data packets and there is a huge load on base stations to manage that incoming and outgoing traffic so communication may be disturbed. On the contrary, if the clustering approach is used then only CHs communicate with the base stations from each cluster thus this use of CHs optimizes the channel contention mechanisms. Clustering is performed through evolutionary algorithms. The idea of evolutionary algorithms states that from the given population of individuals the fittest one will survive. Some candidate solutions are created based on a maximized function. This maximized function is an abstract measure or threshold; better this measure provides more significant results. Based on this fitness measure, the best candidate solution is chosen which will then be used as a basis for finding the next better solution. Some operations are carried out on these candidate solutions like recombination and mutation. In recombination, a new solution (child) is created by applying an operator to two candidate solutions (parents) while in mutation new solution is created using a single candidate solution by applying mutation. This process continues iteratively until a sufficient solution is identified. Through this process, we tend to move towards the most optimal solution.

### 3.1 Work flow of evolutionary algorithms

**Fig 1:**
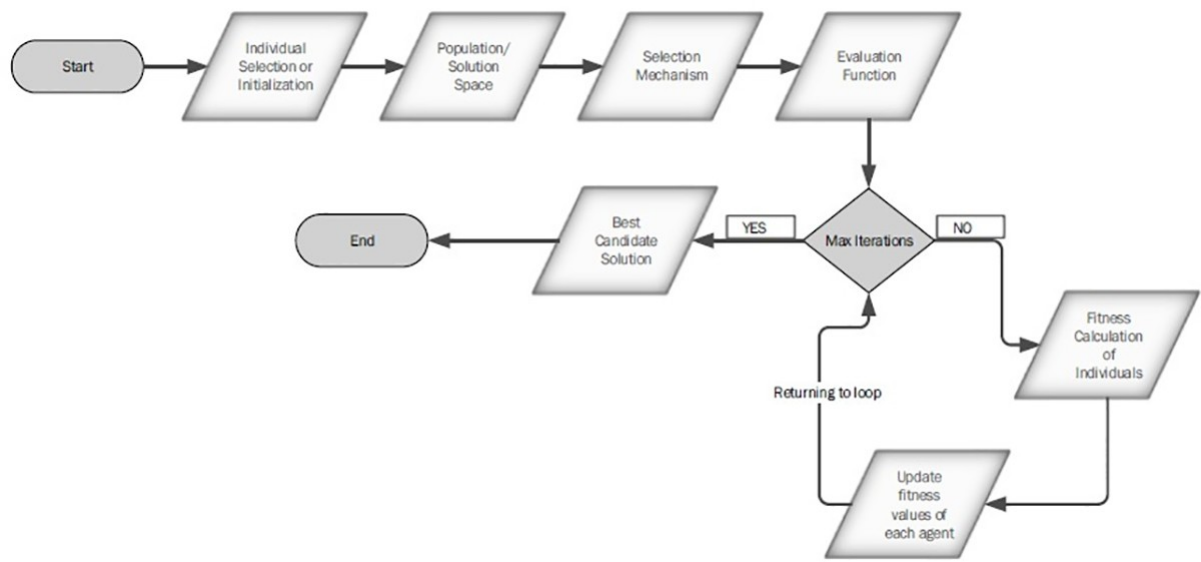
Proposed methodology.

Evolutionary algorithms have components that need to be incorporated while defining these algorithms as shown in Fig 1.

Varies components of the proposed framework as mentioned in Fig 1 are as following:

1. Representation: In representation individuals within evolutionary algorithms are defined.
2. Evaluation Function: A fitness function or maximized function is identified which serves basis for facilitating improvements. This is threshold value and must meet to measure solutions validity.
3. Population: It holds all the possible solutions.
4. Parent Selection Mechanism: Solutions that can become base or parent for the next generation are identified.
5. Variation Operators: Two variation operator mutations and recombination are used to select the new solutions from the old ones.
6. Survivor Selection Mechanism: This is just the same as parent selection, but this is carried out in the next cycle of evolution when the child is ready for evaluation. Child solutions that are capable and most optimizing solutions are replaced with the parents. Now, these children will serve as maximizing functions for next coming solutions.

### 3.2 Whale optimization algorithm

**Fig 2:**
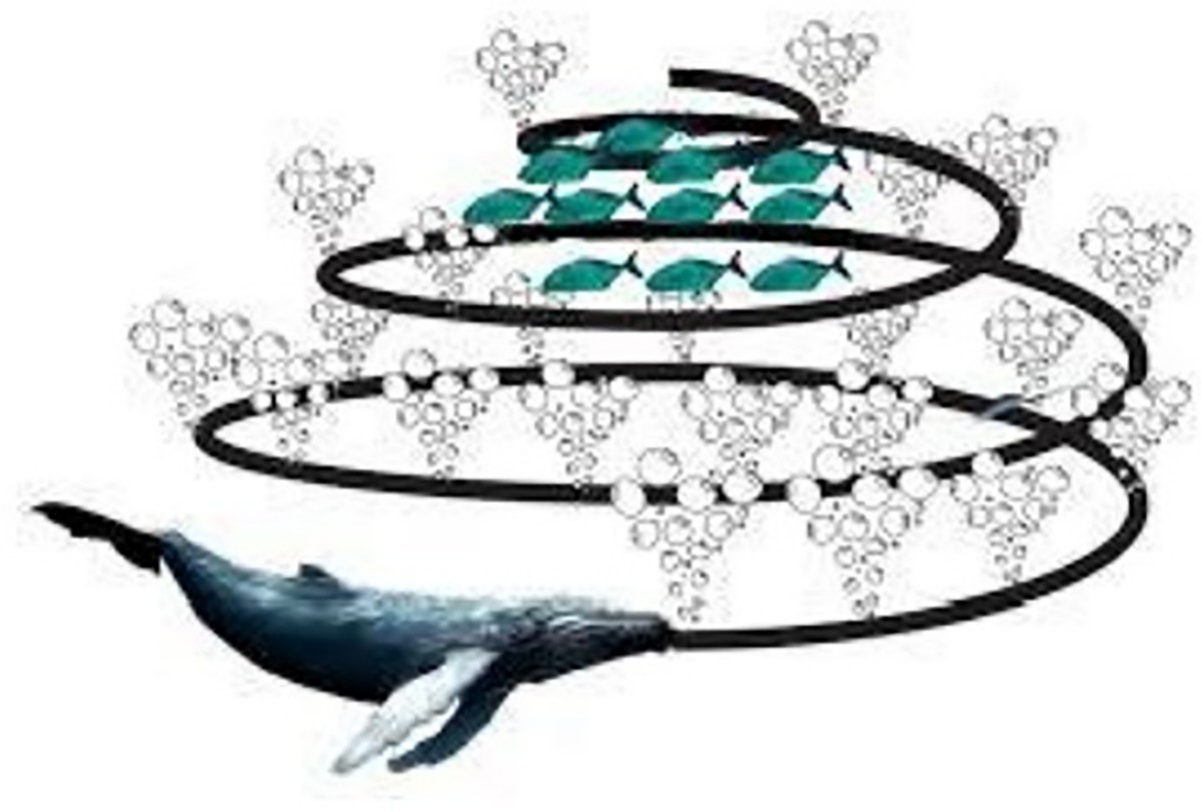
Humpback whales making bubble nets [37].

Whales are known to be fancy aquatic entities. They are observed to be the world’s biggest mammals. A mature whale continues to grow up to 30m in length and the weight is found to be 180T approximately. Major species of whale are seven in number which includes Killer, Minke, Right, Finback, Blue, Sei, and Humpback as shown in Fig 2.

Whales are categorized as hunters in most cases. These giant mammals barely sleep as their breathing mechanism is directly associated with the ocean. They show up on the surface of oceans when breathing. An interesting fact detected about whales is that half portion of the brain is occupied for sleeping purposes. What is more interesting is that these are super-intelligent creatures with emotions as well. As per a study conducted in [37], it is evident that whales possess chambers in particular portions of their brains that are almost identical to spindle cells in humans. Spindle cells found in the human brain are usually accountable for decision making, judgments, emotional fluctuations, and other social behaviors. It is correct to say that spindle cells in human beings are distinctive in comparison to other living beings and distinguish them from other creatures. Whales are found to have two times of chambers in number than a mature humanoid. This is the principal reason for sharpness and cleverness in whales. Studies have proved that a whale might contemplate, learn, decide, interconnect, judge, and also have sentiments. Killer whales can even establish their dialect. However, all emotional states and smartness are on a lower level, if the comparison is to be made with humans. These whales normally hunt group of krill or tiny fishes which are closer to the water surface. Their hunting behavior is termed as bubble-net feeding technique. This is foraging behavior and it is performed by making particular kinds of bubbles in a ring or 9-shaped path as represented in Fig 2. [37] scrutinized this behavior by exploiting tag sensors. They recorded 300 feeding events which were tag-derived bubble-net of nine different humpback whales. Two activities related to bubble were found and they were named as ‘upward spirals and ‘double loops. In the first activity, humpback plunge 12 m deep and then makes circle-shaped bubbles surrounding the target. After that, they swim to the water surface. The second activity has three stages named coral spiral, lobtail and catch circle. It is noteworthy that bubble-net feeding is a distinctive action that is seen only in humpback whales.

### 3.3 WOACNET mathematical modelling

In this research work, a spiral bubble-net feeding operation is numerically and scientifically demonstrated for performing the optimization. This section deals with mathematical modeling of enclosing target, loop bubble-net feeding plotting, and target search as follows:

#### 3.3.1 Encircling prey

Humpback whales sense the position of their targeted victim and encircle them. As the location in the search space is not known for the optimal design a priori, the WOACNET suppose that the present best candidate solution is the target victim or is close to the optimal. Once the optimized exploration is established, other exploration agents apprise their locations according to the optimized search agent as shown in Eqs (1) and (2).

$$\vec{D}=|\vec{C.}{\vec{X}}^{*}(t)-\vec{X}(t)|$$ (1)

$$\vec{X}(t+1)=\vec{X^{*}}(t)-\vec{A}.\vec{D}$$ (2)

Here t shows existing repetition, $\vec{A}$ and $\vec{C}$ are coefficient vectors, X* shows position vector of the most optimal solution found so far, $\vec{X}$ is the position vector, | |is an absolute value, and”.” is the element by element multiplication. X* must be updated during every iteration if a better solution is found. The vectors $\vec{A}$ and $\vec{C}$ can be calculated as per Eqs (3) and (4):

$$\vec{A}=2\vec{a}.\vec{r}–\vec{a}$$ (3)

$$\vec{C}=2.\vec{r}$$ (4)

**Fig 3:**
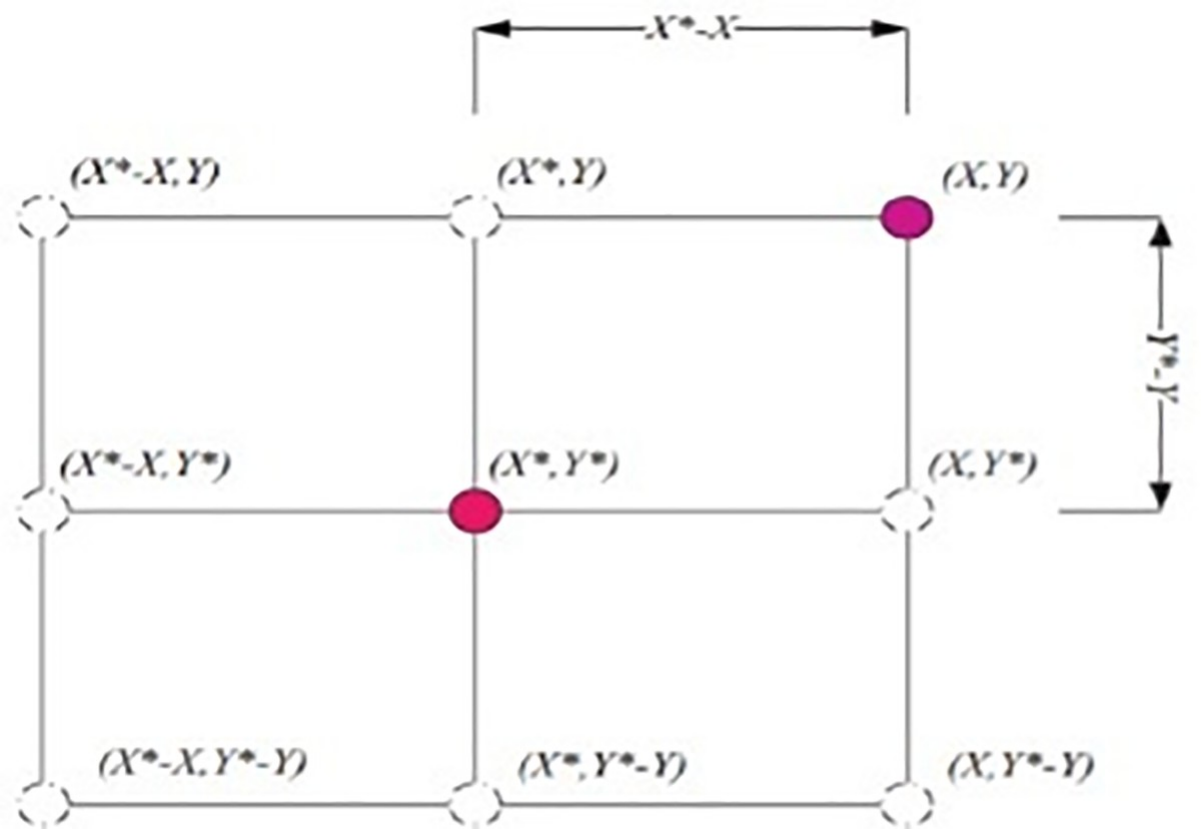
Position vectors along with possible next locations in two dimensions (2D) [37].

$\vec{a}$ is minimized from 2 to 0 during iterations in exploration and exploitation phases and where $\vec{r}$ is random vector in [0,1]. Fig 3 shows the justification required Eq (2) for the two-dimensional problem.

**Fig 4:**
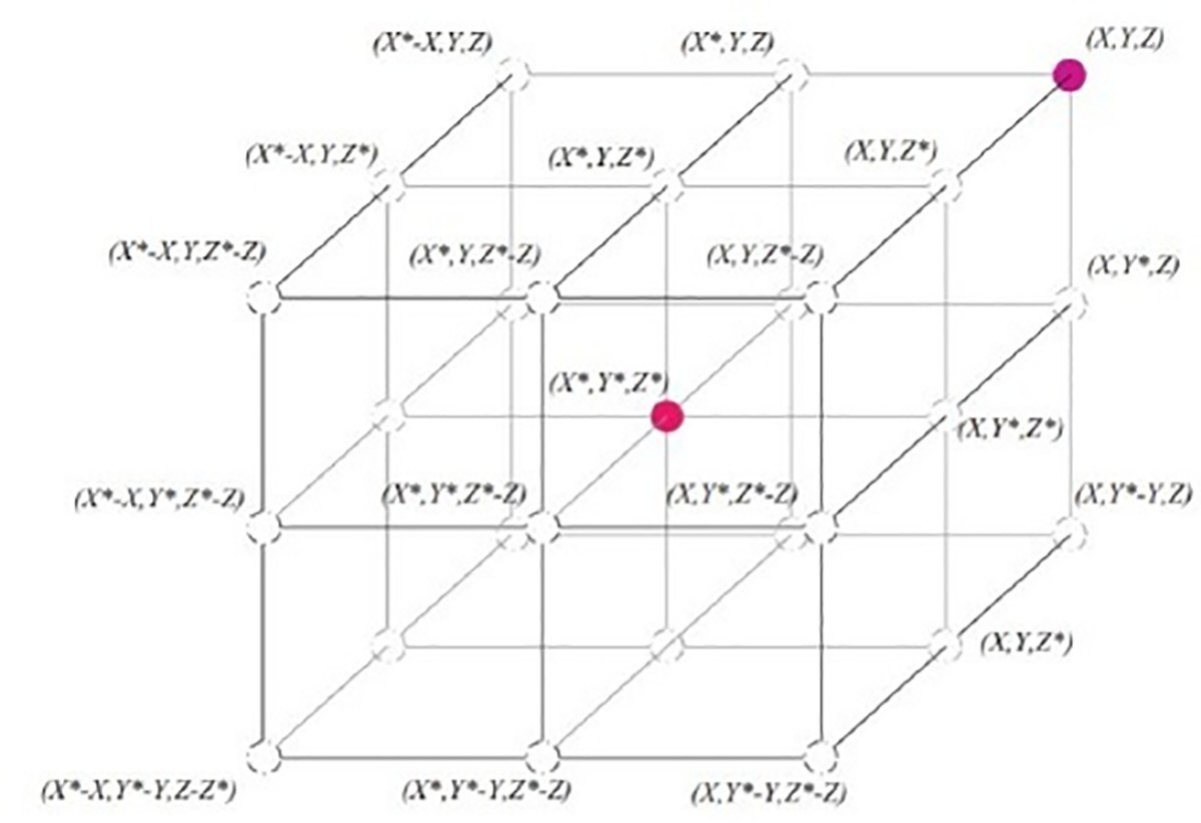
Position vectors along with possible next locations in three dimensions (3D) [37].

The location of (X, Y) of the search agent can be updated by the location of the best record (X*Y*) which is currently found. Various positions around the best agent can be obtained for the current location by managing the values of vectors A⃗ and C⃗. Possibly found updating position of the search agent in three-dimensional space is presented in Fig 4.

Any point of exploration universe sited among key-points discussed in Fig 4 can be reachable once the random vector (r)→ is determined. That is why it can be seen that Eq (2) permits each search agent to update its location in the region of the present optimal result and further simulates encompassing the target. Extension of this approach is implied to search space having “*n”* dimensions and search agents continue their movement in hyper-cubes around optimal solution acquired till that time. It has been mentioned previously that humpback whales also hunt their prey with bubble-net strategy and this approach is mathematically calculated in next section.

#### 3.3.2 Bubble-net attacking method (exploitation phase)

Two methods are suggested for mathematical modeling of bubble-net behavior shown by humpback whales.

**Fig 5:**
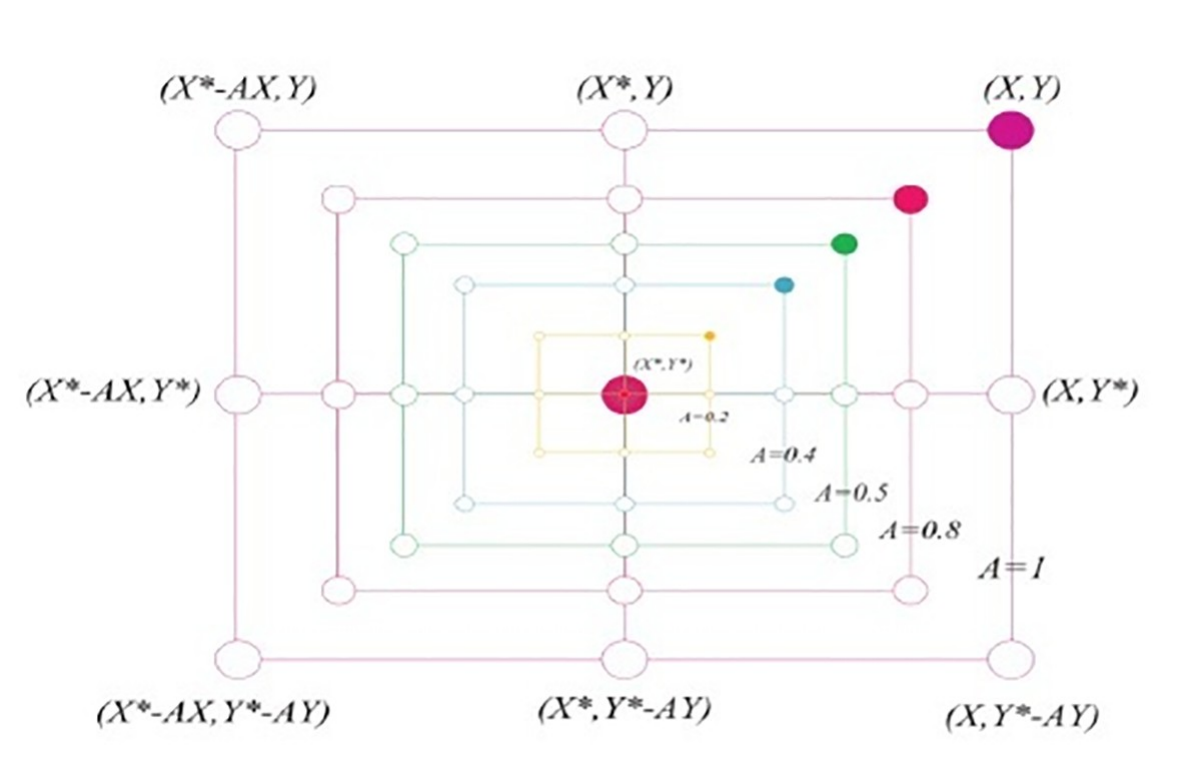
Shrinking encircling mechanism in bubble-net search [37].

*3*.*3*.*2*.*1 Shrinking encircling mechanism*. This behavior is attained by minimizing the value of $\vec{a}$ in Eq (3). The range to which $\vec{A}$ is fluctuated is also minimized by $\vec{a}$. It can be said that $\vec{a}$ is a random value in the interval [-a, a] where a is minimized from 2 to 0 over a certain set of iterations. Setting random values for $\vec{A}$ in [–1,1], the new position of a search agent can be determined anywhere among the actual position of the agent and the position of the current best agent. Fig 5 represents attainable positions from (X, Y) to (X*, Y*) which are reached by **0≤A≥1** in a two-dimensional space.

*3*.*3*.*2*.*2 Spiral updating position*. This technique calculates the distance between the whale’s location (X, Y) and the prey’s location (X*, Y*). Afterward, a spiral equation is created between the two positions to depict the helix-shaped movement of humpback whales as mentioned in Eq (5):

$$\vec{X}(t+1)=\vec{D^{\prime }}.e^{bl}.\cos\left(2\pi l\right)+\vec{X*}(t)$$ (5)

Here $\vec{D^{\prime }}=|\vec{X*}(t)-\vec{X}(t)$ and it shows the distance of ith whale to prey (most optimal solution attained so far), b is considered as a constant to determine the shape of a logarithmic spiral, l is a random number in [–1,1], and “.” is an element by element multiplication. Humpback whales swim in the surroundings of their targeted prey in a circle and spiral-shaped path as well. They show two simultaneous behaviors and to model it, we suppose that there exists a probability of 50% of both actions being exhibited by them during optimization as mentioned in Eq (6):

$$\vec{X}(t+1)=\left\{ \begin{array}{c} \vec{X*}(t)-\vec{A}.\vec{D}\;if\;p<0.5\\ \vec{D\prime }.e^{bl}.\cos\left(2\pi l\right)+\vec{X*}(t)\;if\;p\geq 0.5 \end{array}\right.$$ (6)

Where p is a random number in [0, 1]. Humpback whales also search out for prey randomly, and the mathematical model is presented in the following section.

#### 3.3.3 Search for prey (exploration phase)

Similar mechanism based on a modification of vector $\vec{A}$ can be exploited for prey searching (exploration). Humpback whales also perform random searches following their respective positions. To get the search agent in moving the state far from the reference whale $\vec{A}$ has been used with random values between 1 and -1.

Unlike the exploitation phase, the location of the search agent is updated in this stage about the randomly picked agent. This approach and $|\vec{A}|>1$ focus on investigation and permit the WOACNET to conduct a global search. The mathematical model is mentioned in Eq (7):

$$\vec{D}=|\vec{C}.\vec{X_{rand}}-\vec{X}|$$ (7)

This algorithm begins with a set of random possibilities. In all iterations, search agents update their locations with either a randomly picked search agent or the best solution acquired till that time. The factor is minimized from 2 to 0 so that searching and preying can be accommodated. A random search agent is picked when $|\vec{A}|>1$. However, the most optimal solution is chosen when $\vec{\left|A\right|}<1$ for updating the locations of search agents. WOA can switch among spiral and bubble-net attacking maneuver as shown in Eq (8).

$$\vec{X}(t+1)=\vec{X_{rand}}-\vec{A}.\vec{D}$$ (8)

Where $\vec{X_{rand}}$ is a random location vector selected from the existing population. Some probable positions of a specific solution with $\vec{A}>1$ as previously mentioned in Fig 5 circular movement depending on the value of p for a section of an optimal number of clusters. The mathematical modeling and simulation for the developed WOACNET based method is represented in the form of pseudo-code as mentioned in Table 1:

**Table 1:** Pseudocode of developed WOACNET.

Pseudocode of developed WOACNET
1: Initialize each vehicle, along with position, direction and the speed of each vehicle on highway 2: Create a mesh topology among nodes/vertices where each vertex represents the vehicle id 3: Initialize same search agent values for each edge for the above mesh topology 4: Calculate distance of each vehicle with others, normalize and associate these distance values with the corresponding edges in the above mesh topology. 5: X* = the best search agent (Cluster Head) 6: **While** (current iteration< maximum number of iterations) **for** each search agent Update a, A, C, l, and p **if1**(p<0.5) **if2**(|A|< 1) Update the position of the current vehicle by $\vec{D}=|\vec{C.}{\vec{X}}^{*}(t)-\vec{X}(t)|$ **else if2**(|A| ≥ 1) Select a random search agent (Xrand) Update the position of the current vehicle by $\vec{X}(t+1)=\vec{X_{rand}}-\vec{A}.\vec{D}$ **end if2** **elseif1(p ≥ 0.5)** Update the position of the current vehicle by $\vec{X}(t+1)=\vec{D^{\prime }}.e^{bl}.\cos\left(2\pi l\right)+\vec{X*}(t)$ **end if1** **end for** 7: Check if any search agent goes beyond the search space and amend it 8: Calculate the fitness of each vehicle 9: Update X* if there is a better solution Current iteration = current iteration+1 10: **end while** 11: return X*

From a conceptual perspective, the proposed WOACNET is a global optimizer as it incorporates both, searching and preying capabilities. Moreover, the proposed hyper-cube technique determines a search in the region of the optimal result and allows other search agents to make use of the best result at present in that domain. Adaptive varying of search vector A allows WOACNET to transit between searching and preying (by minimizing A, some repetitions are specified for searching $|\vec{A}|\geq 1$ and others are for exploitation $\vec{\left|A\right|}<1$) without any difficulty.

It is also noteworthy that WOA has only two major parameters that require adjustment A in Eq (3) and C in Eq (4). In this study, the volume of heuristics and the set of instances are minimized therefore, ended up implementing the basic category of the WOACNET.

## 4. Results and discussion

In this section, the results are presented from diverse perceptions like grid size, transmission range, and a number of nodes. Subsequent to modeling, simulations were performed for various grid sizes. The results were compared with ALO, GWO, and WOA. The number of clusters was generated synthetically against transmission ranges from 100m to 660m and considering grid size of 1000m x 1000m. The simulation parameters have been presented in Table 2.

**Table 2:** Simulation parameters.

Parameters	Values
**Population Size (Particles)**	100
**Maximum Iterations**	150
**Inertia Weight W**	0.694
**Lower Bound (lb)**	0
**Upper Bound (ub)**	100
**Dimensions**	2
**Transmission Range**	100m-600m
**Mobility Model**	Random Waypoint
**Simulation Runs**	10
**W**_**1**_ **(weight of first objective function) (Multi-objective)**	0.5
**W**_**2**_ **(weight of second objective function) (Multi-objective)**	0.5
**Nodes**	20–50

**Fig 6:**
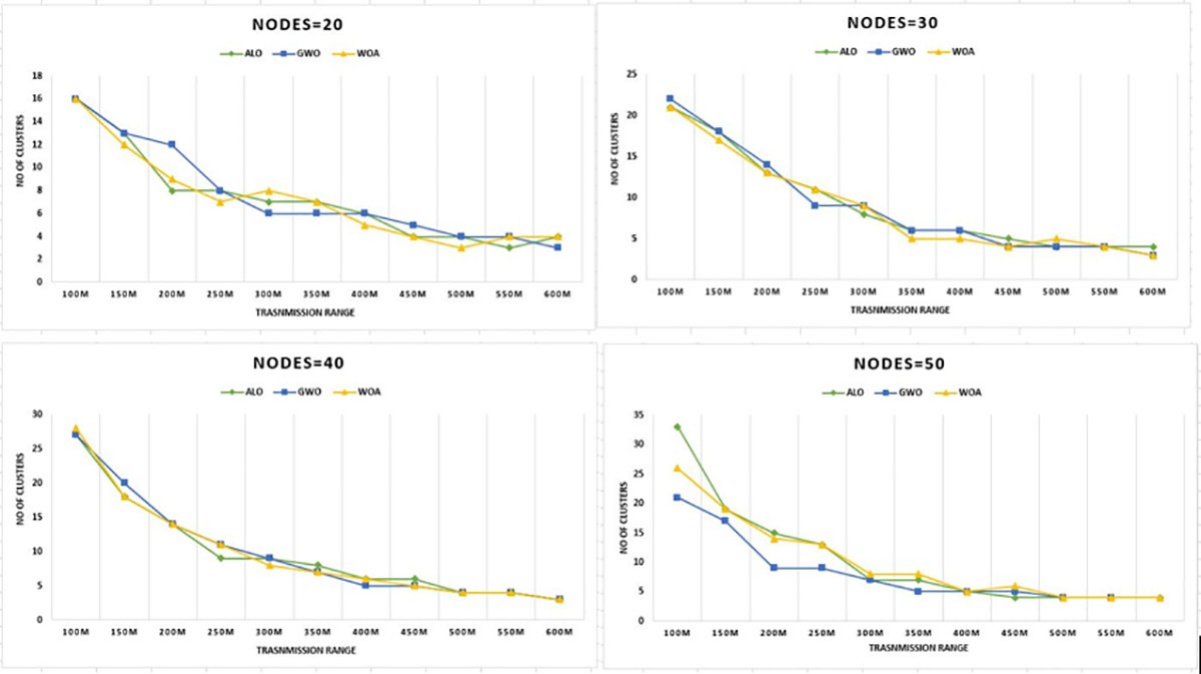
Transmission range versus CHs for nodes 20–50 & grid size 1km x 1km.

Next, to test the capability of the developed method simulations were also carried out for a diverse number of nodes 20, 30, 40, and 50. The WOA was performing well at minimum cost for all groups of vehicles. The results in Fig 6 show the superiority in terms of cost reduction for various transmission ranges.

**Fig 7:**
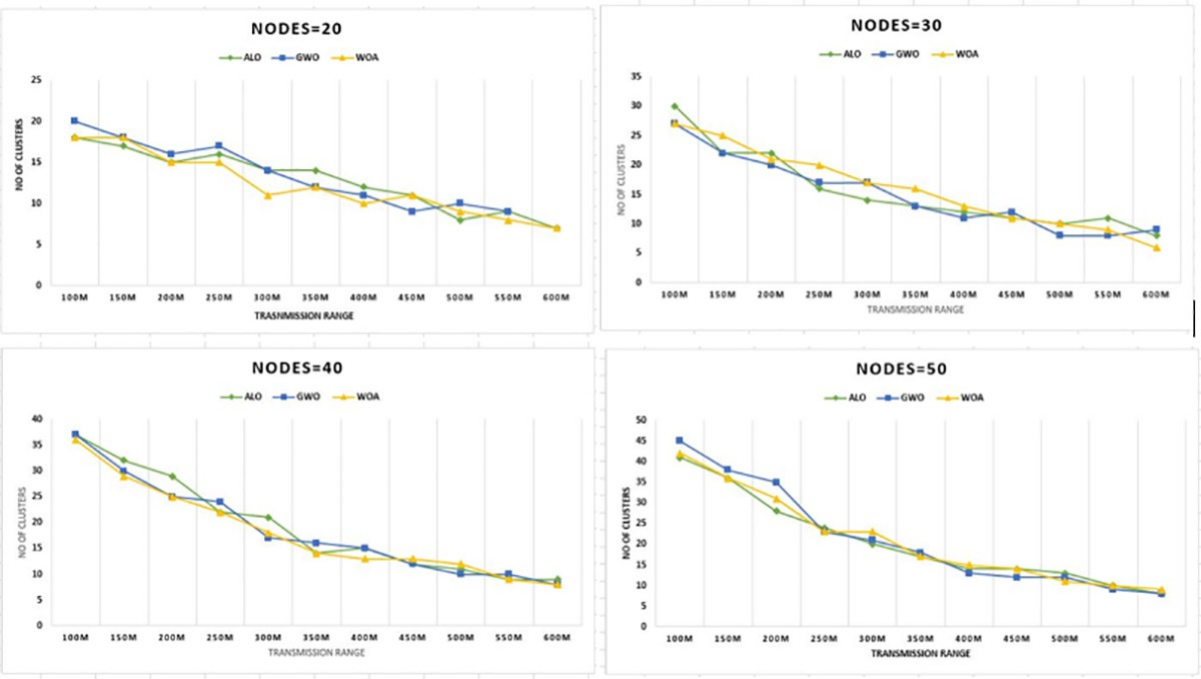
Transmission range versus CHs for nodes 20–50 & grid size 2km x 2km.

The next result presented in Fig 7 is for 2000m by 2000m grid size. These results also show the proposed WOA is the most cost-effective algorithm for communication. During experimentation, a distinctive relationship which is, increasing the transmission range result in a decrease in the number of clusters.

**Fig 8:**
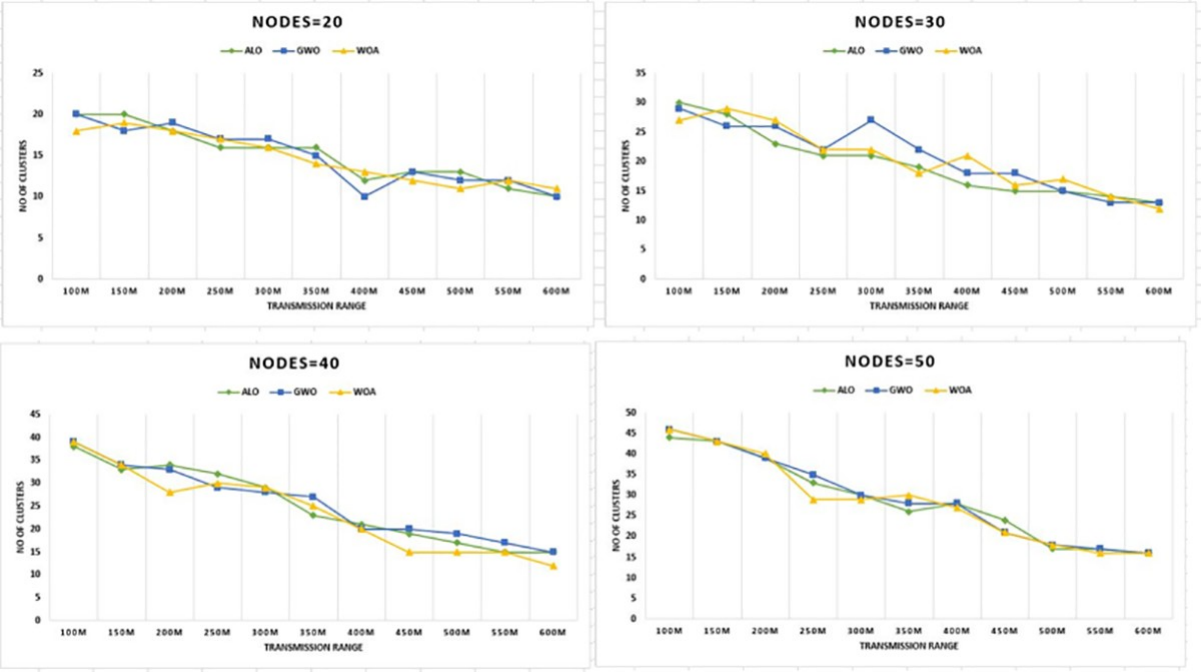
Transmission range versus CHs for nodes 20–50 & grid size 3km x 3km.

The parameters for communication range and number of clusters are contrariwise related to each other, that means, by decreasing the communication range, the number of clusters of the entire network increase and vice versa. The number of clusters also has an impact on network resources, a rise in the number of clusters increases the required resources. Further, experimentations were conducted considering a grid size of 3Km by 3Km having 20 to 50 nodes. The results presented in Fig 8 show the developed WOACNET performs better compared to other methods for the said scenarios. It is evident from the results that the suggested optimized WOACNET technique optimizes the routing by efficient clustering which reduces the number of hopes for network communication and this ultimately leads to the minimized packet delays and routing cost and this results in less number of resources required for a fewer number of clusters.

**Fig 9:**
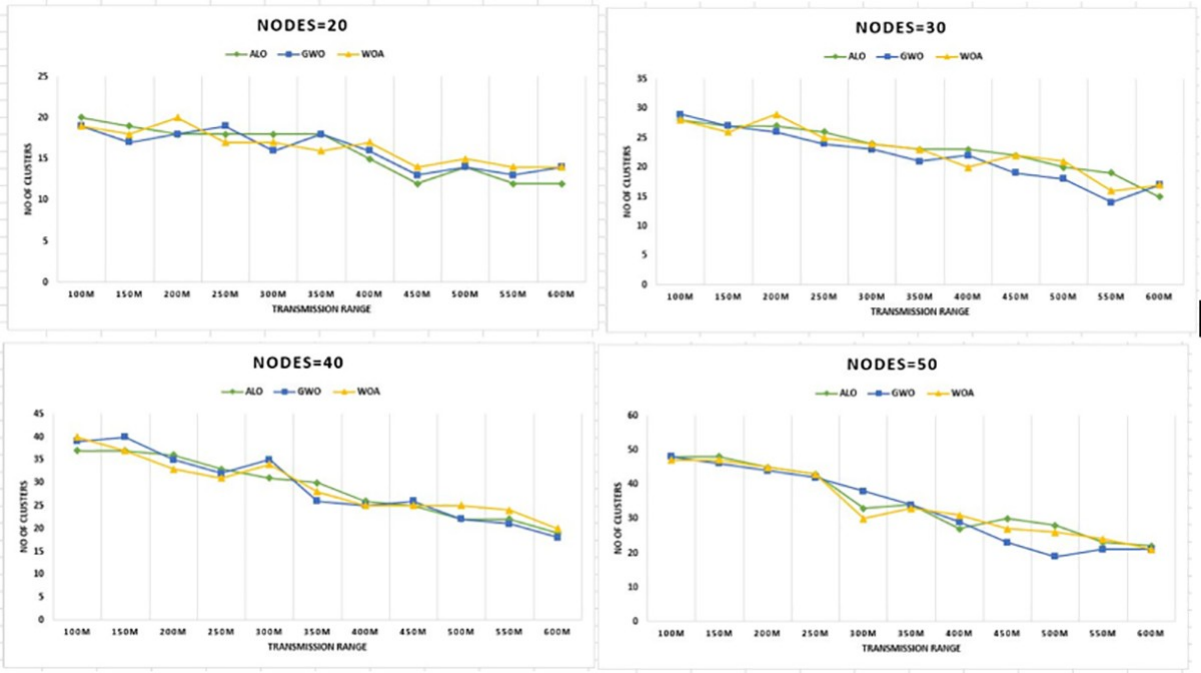
Transmission range versus CHs for nodes 20–50 & grid size 4km x 4km.

Further experimentation was performed for the same parameters except for the value of grid size which is changed to 4Km x 4Km as shown in Fig 9, for 50 number of nodes and at the transmission range of 300 meters WOACNET specifically performs better.

**Fig 10:**
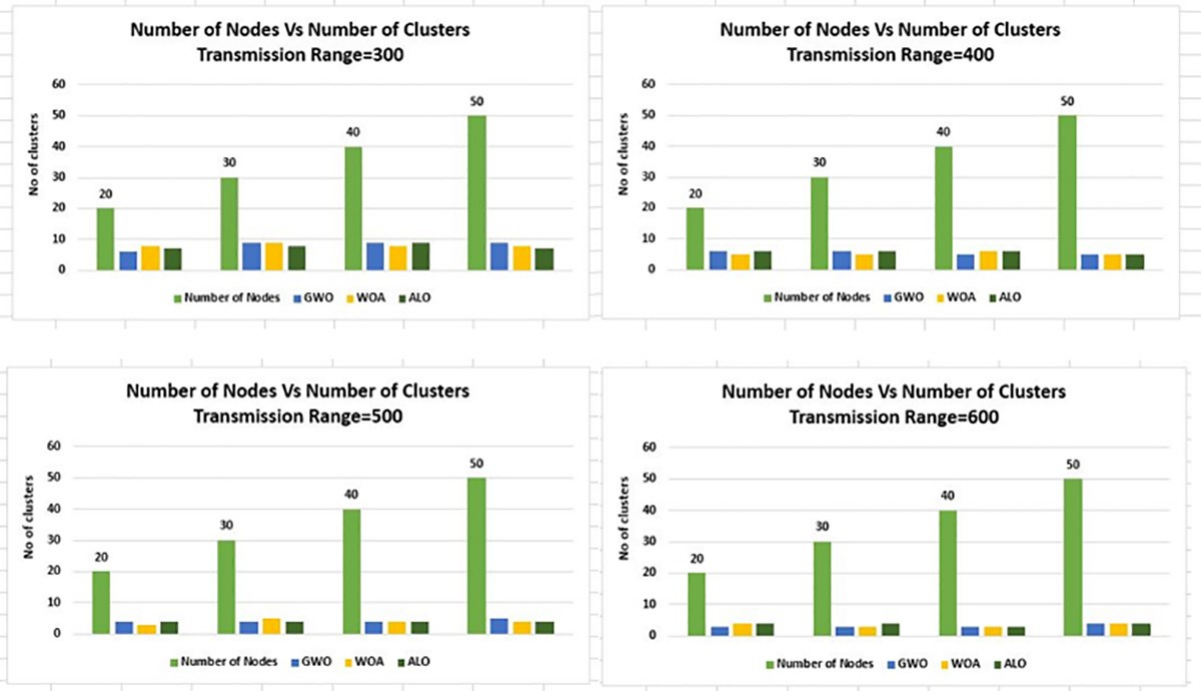
Node density versus number of clusters for transmission range 300m-600m & grid 1Km×1Km.

In Fig 10 the results are laid down from a different perspective for better understanding, in which node density is associated with a number of clusters, the communication range is changed from 300m to 600m and the grid size value is kept static at 1Km×1Km.

**Fig 11:**
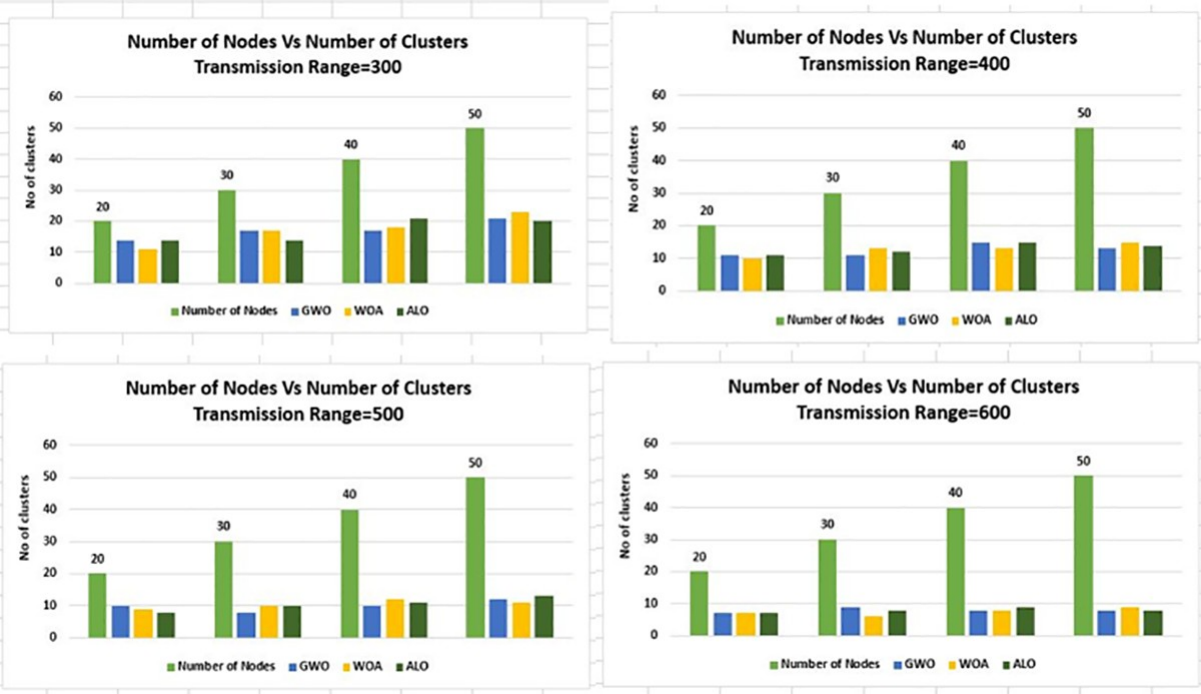
Node density versus number of clusters for transmission range 300m-600m & grid 2Km×2Km.

While in Fig 11 the value of grid size is changed to 2Km×2Km and all other parameters are kept the same as in Fig 10. In Fig 11 the node density versus the number of clusters is presented, the trend is the same as Fig 10.

**Fig 12:**
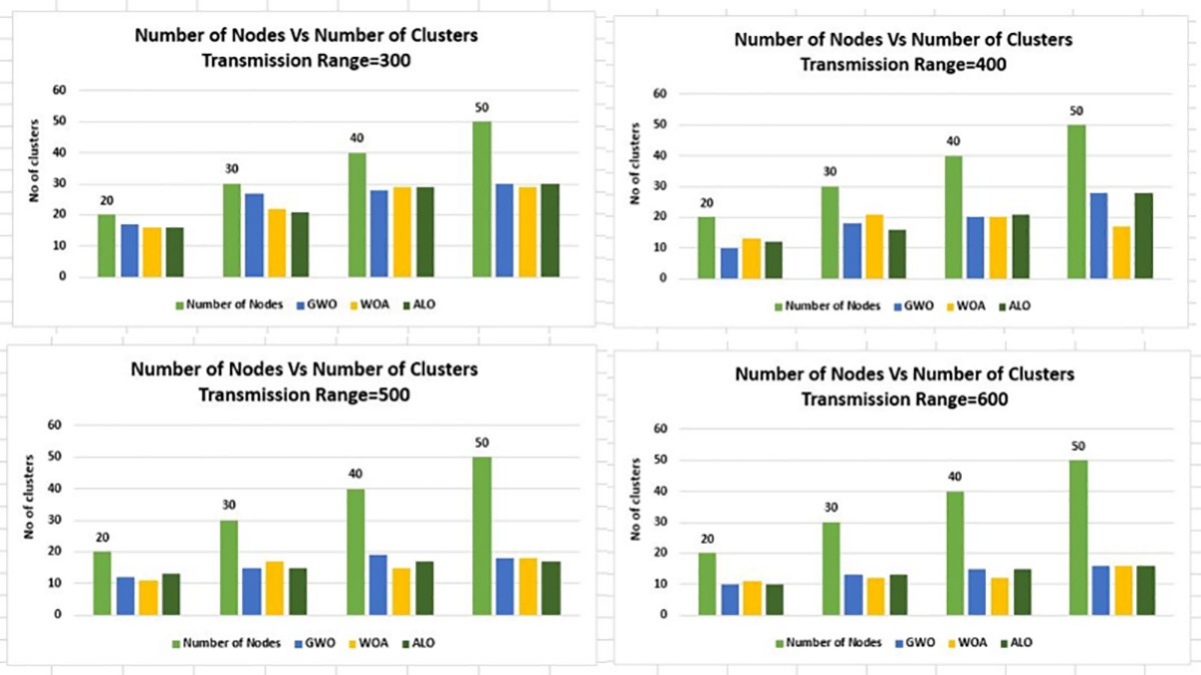
Node density versus number of clusters for transmission range 300m-600m & grid 3Km×3Km.

**Fig 13:**
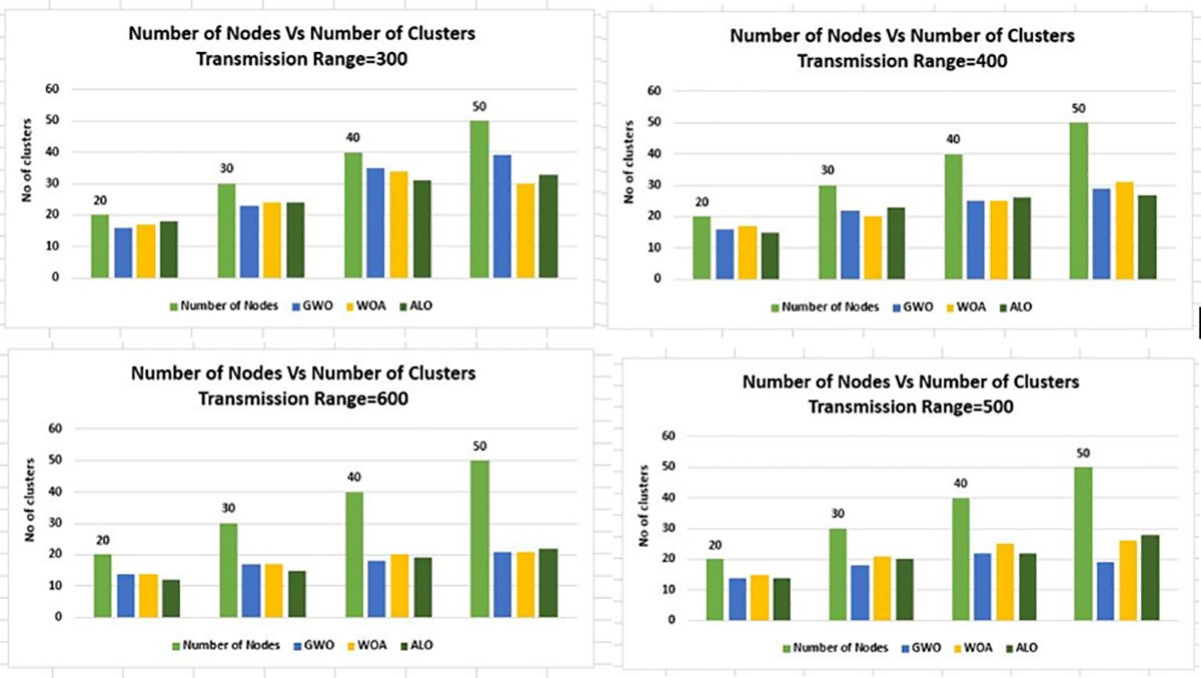
Node density versus number of clusters for transmission range 300m-600m & grid 4Km×4Km.

In Figs 12 and 13 the same experimentation is performed for different grid sizes, i.e. 3Km×3Km and 4Km×4Km respectively.

**Fig 14:**
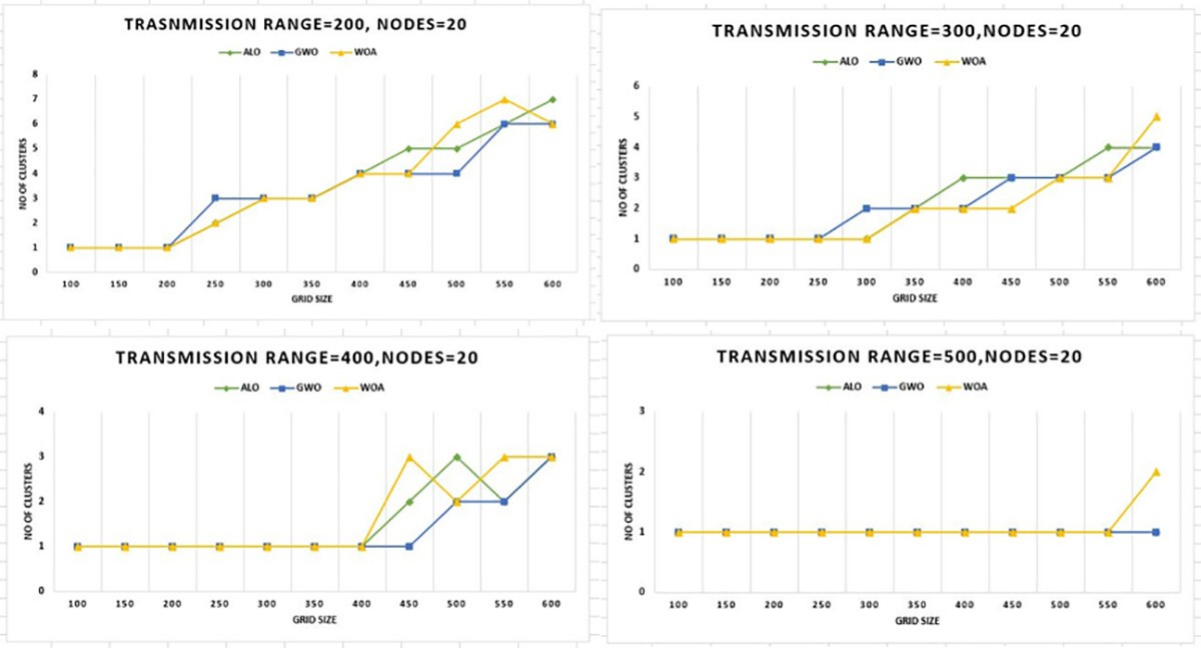
Grid size versus number of clusters for 20 nodes.

**Fig 15:**
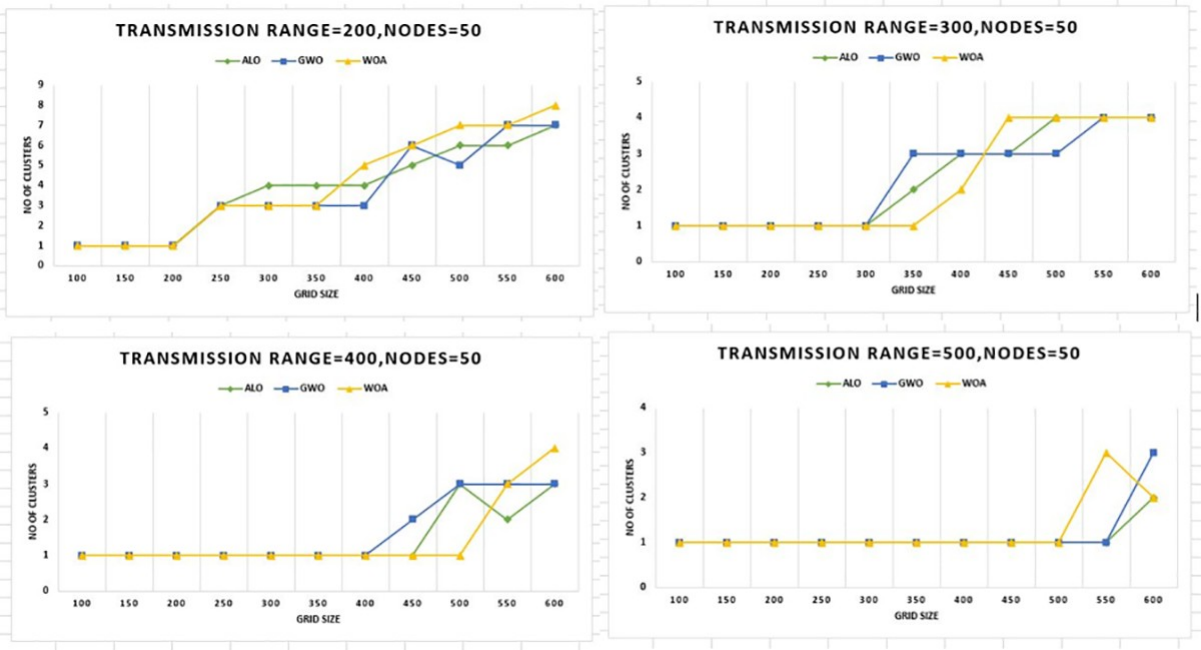
Grid size versus number of clusters for 50 nodes.

Figs 14 and 15 show the results from a completely new perspective. In Fig 14 the number of nodes kept static i.e. 20, and outcomes are compared by keeping the values of grid size at the X-axis and the number of clusters at the y-axis. Similarly, the same experimentation is performed while keeping the number of nodes 50, it is evident that WOACNET performs better as compared to the other algorithms from this perspective as well.

### 4.1 Load Balance Factor (LBF)

**Fig 16:**
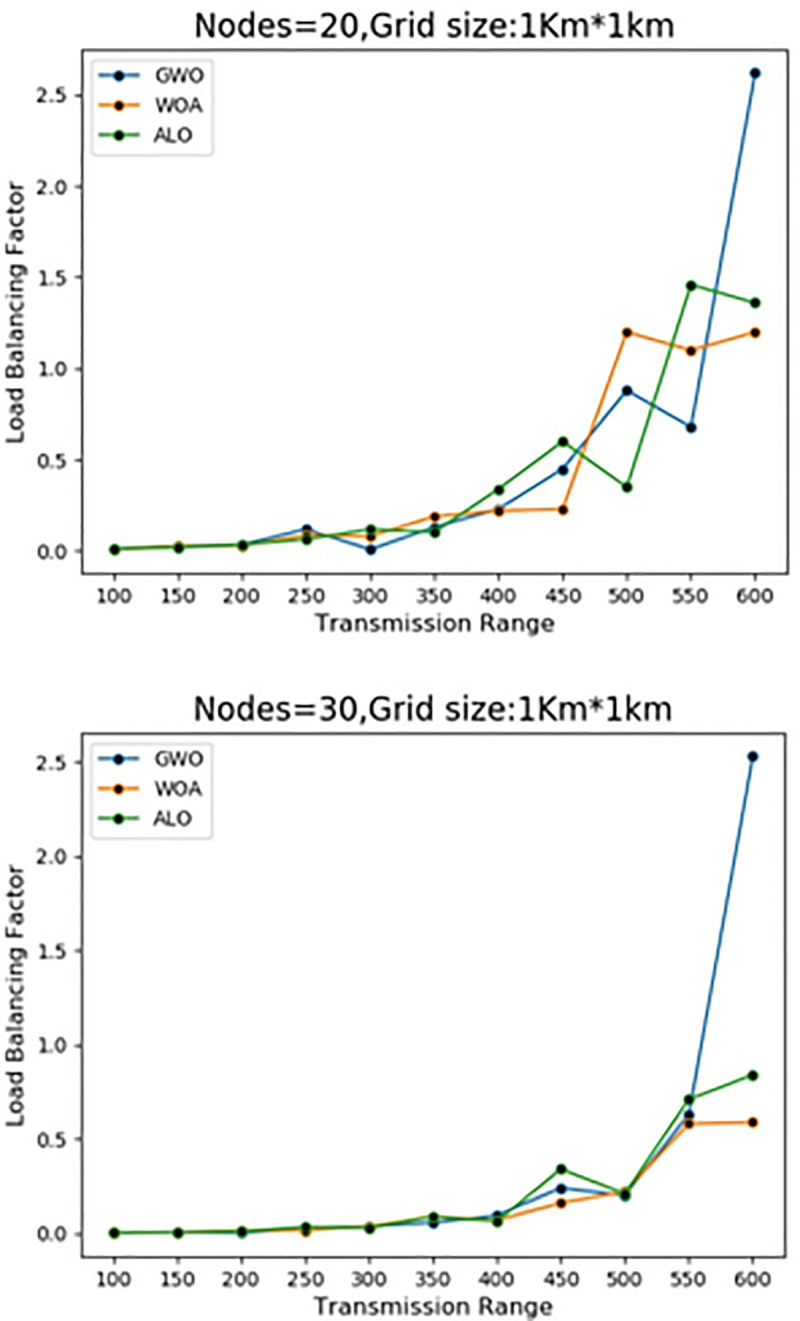
Load balance factor for 20 & 30 nodes at the grid size of 1Km x 1Km.

In literature, LBF is usually incorporated as an assessment tool relating to any technique and to measure Cluster Head load [20, 23]. Ideally, each Cluster Head should deal with an equivalent number of Cluster Nodes, yet it is extremely hard to keep a consummately load-adjusted framework consistently. The fundamental explanation is the successive separation and connection of neighbors from the Cluster Heads. The elements of the cluster size show the load of a Cluster Heads. The Load Balanced Factor is defined in Eq (9),

$$LBF=\frac{1}{nc*\Sigma i(xi\_\mu )2}$$ (9)

where nc is the number of Cluster Heads, xi is the nodes of cluster i, and _ *μ* = N − nc/nc is the average number of groups of a Cluster Head (being the total number of nodes in the system). Fig 16 shows that WOACNET is performing better as the number of nearest nodes approaches its maximum value than ALO and GWO regarding adjusting the load in the network. All the experiments are performed and the results of the suggested algorithm is evaluated against the other methods for the fallout.

### 4.2 Statistical tests and analysis

To further explore the capabilities of the developed method, various statistical tests such as p-test, regression analysis, and ANOVA were performed for validation. The results are illustrated in Table 3.

**Table 3:** Regression coefficients of transmission range on no of clusters.

	Variable	*B*	p-value	β	*SE*	R^2^	ANOVA (F-value
WOACNET (This study)	Constant	14.800***	0		1.312	0.87	56.8***
	TR	-0.022***	0	-0.924	0.003		
GWO [23]	Constant	15.880***	0		1.214	0.863	40.1***
	TR	-0.020***	0	-0.919	0.003		
ALO [20]	Constant	14.836***	0		1.301		38.8***
	TR	-0.021***	0	-0.901	0.003	0.812	

***P < 0.01.

*P < 0.05.

*P < 0.1.

Transmission Range (TR) Is the Independent (Predictor) variable.

No of Clusters is the dependent (Outcome) variable.

Table 3 shows the impact of the transmission range on the number of clusters in the case of WOACNET, GWO, and ALO. As per the theory of study the more the range of transmission the lesser will be the number of clusters. In the case of WOACNET, the study examined that a 1% increase in the transmission range will decrease the number of clusters by .022%. In comparison, the other two state-of-the-art techniques i.e., GWO and ALO the change in TR brings less decrease i.e., 0.020 and 0.021 respectively. The findings revealed that in all the state-of-the-art techniques the Transmission Range negatively predicts the No of clusters with β = -0.924***, -0.919***, and -0.901*** respectively. The R^2^ value shows the predictor variable explained 0.87%, 0.86%, and 0.81% variance respectively in the outcome variable i.e. No of clusters with F (1, 9) = 56.8***, F (1, 9) = 40.1*** and F (1, 9) = 38.8***

Table 4 shows the impact of grid size on the number of clusters in the case of WOACNET, GWO, and ALO. As per the theory of study the more the grid size the larger will be the number of clusters. In the case of WOACNET, the study examined that 1% increase in the Grid Size will increase the number of clusters by 0.013%. In comparison, the other two state-of-the-art techniques i.e., GWO and ALO the change in Grid Size brings less increase i.e., 0.011% and 0.012% respectively.

**Table 4:** Regression coefficients of grid size on no of clusters.

	Variable	*B*	p-value	β	*SE*	R2	ANOVA (F-value
WOACNET (This study)	Constant	-0.936***	0.00		0.453	0.926	113.05***
	GS	0.013***	0.00	0.962	0.001		
GWO [23]	Constant	-0.355***	0.00		0.397	0.918	100.48***
	GS	0.011***	0.00	0.958	0.001		
ALO [20]	Constant	-0.930***	0.00		0.294	0.922	112.61***
	GS	0.012***	0.00	0.960	0.001		

***P < 0.01.

**P < 0.05.

*P < 0.1.

Grid Size (GS) Is the Independent (Predictor) variable.

No of Clusters is the dependent (Outcome) variable.

The findings revealed that in all the state-of-the-art techniques the Grid Size positively predicts the No of clusters with β = 0.962***, 0.958*** and 0.960*** respectively. The R^2^ value shows the predictor variable explained 0.926%, 0.918%, and 0.922% variance respectively in the outcome variable i.e. No of clusters with F(1, 9) = 113.05***, F(1, 9) = 100.48*** and F(1, 9) = 112.61***.

Table 5 shows the impact of the transmission range on the load balance factor in the case of WOACNET, GWO, and ALO. As per the theory of study the more the transmission ranges the larger will be the load balance factor. In the case of WOACNET, the study examined that a 1% increase in the TR would increase the LBF by 0.003%. In comparison, the other two techniques i.e., GWO and ALO the change in GS brings less increase i.e., 0.001% and 0.002% respectively.

**Table 5:** Regression coefficients of transmission range on load balance factor.

	Variable	*B*	p-value	β	*SE*	R2	ANOVA (F-value
WOACNET (This study)	Constant	-0.523**	0.02		0.192	0.779	31.64***
	TR	0.003***	0.00	0.882	0.001		
GWO [23]	Constant	-0.683*	0.09		0.370	0.571	11.95***
	TR	0.001***	0.00	0.755	0.001		
ALO [20]	Constant	-0.534**	0.04		0.294	0.712	22.50**
	TR	0.002***	0.00	0.845	0.001		

***P < 0.01.

**P < 0.05.

*P < 0.1.

Transmission Range (TR) Is the Independent (Predictor) variable.

Load Balance Factor (LBF) is the dependent (Outcome) variable.

Table 5 shows the impact of the transmission range on the load balance factor in the case of WOACNET, GWO, and ALO. As per the theory of study the more the transmission ranges the larger will be the load balance factor. In the case of WOACNET, the study examined that a 1% increase in the TR will increase the LBF by 0.003%. In comparison, the other two techniques i.e., GWO and ALO the change in GS brings less increase i.e., 0.001% and 0.002% respectively. The findings revealed that in all the state-of-the-art techniques the TR positively predicts the No of clusters with β = 0.882***, 0.755*** and 0.845*** respectively. The R^2^ value shows the predictor variable explained 0.779%, 0.571%, and 0.712% variance respectively in the outcome variable i.e., LBF with F (1, 9) = 31.64**, F (1, 9) = 11.95*** and F (1, 9) = 22.50**.

Figs 6–16 shows that WOA considerably show improved grades as equated to other mentioned algorithms. The results justify the relationship between the communication range and the number of clusters, the vital resources for the number of clusters. This optimization ultimately reduces the routing cost for the network. The results in Fig 6 shows that WOACNET forms fifteen clusters initially and then moves to fifty-four clusters for sixty nodes which shows better performance in terms of optimized clusters for transmission range of 1 meter. The detailed and experimentation and analysis for the 2000 m by 2000 m grid shows in Fig 7 that the proposed WOACNET performs better as compared to other mentioned algorithms. The results for the 4000 m by 4000 m grid for 100m to 600m transmission ranges are presented in Fig 9. It is evident from Figs 8 and 9 that number of clusters increases by increasing grid size. This shows of grid size and the number of clusters are directly related. Further experimentations were conducted with 2000 m by 2000 m grid size and taking transmission range from 100m to 600m. The distance between nodes increases by increasing the grid size which shows a direct relation and ultimately increases in grid size isolate the nodes. The increase in a high number of isolated nodes results in the maximum number of clusters for each mentioned scheme. It is observed from Figs 6 and 7 that the methods GWO and WOA produced slightly the same number of clusters. However, the proposed WOACNET still performs better as compared to ALO and reduces clusters by 46%. Therefore, it is accomplished that the relationship between the communication range and the number of clusters is contrariwise proportional. Therefore, the number of clusters decreases by increasing the communication range hence results in a large number of clusters are required to cover a large area. It was found that for combinatorial optimization problems like clustering maintaining a state of equilibrium between the various exploratory and exploitative phases provides a better optimal solution. It was further observed that the developed method WOACNET produced a considerably small amount of clusters when compared to other techniques. This study optimizes several clusters intelligently and efficiently in Vehicular Ad Hoc Network and, the overlap of the developed WOACNET and other techniques on few occasions are due to the random nature of evolutionary algorithms.

## 5. Conclusion

There are various VANETs techniques suggested in the literature for optimizing the utilization of the resources. The available resources in VANETs are limited as compared to the other networks. Therefore, the efficient utilization of these limited resources is necessary. Clustering, a technique for resource optimizing, cluster optimization schemes are also available in the literature. In this paper, a bio-inspired node clustering optimized approach is implemented, which is inspired by the nature of whales. A performance evaluation and analysis of this algorithm with the modern and advanced schemes is presented. The proposed scheme (WOACNET) performs better (46%) than the ALO, and GWO in terms of the number of CHs, while varying transmission ranges, grid size, and several nodes. It reduces the communication cost for the network by minimizing the number of clusters to near optimum and increased cluster lifetime. This minimized number of clusters additionally prompts to fewer resource requirements in VANETs.

In the future, experimentation on extending the developed method for multi-objective functions for rapid changing of vehicle topologies is in progress.

The following abbreviations are used in this manuscript:

## Abbreviations

ALO: Ant Lion Optimization
WOACNET: An Intelligent Cluster Optimization Algorithm based on Whale Optimization Algorithm for VANETs
CH: Cluster-Heads
CN: Cluster nodes
DSRC: Dedicated Short Range Communications
GWOCNET: Gray Wolf Optimization based clustering algorithm
MANET: Mobile ad hoc networks
ITS: Intelligent Transportation Systems
MOP: Multi-objective optimization problem
WSN: Wireless Sensor Networks
PSO: Particle swarm optimization
VANET: Vehicular ad hoc networks
WCA: Weighted clustering algorithm
LBF: Load Balanced Factor
